# Salmagundi

**Published:** 1885-12

**Authors:** 


### SALMAGUNDI.

EDITED BY E. G. BETTY, D. D. S.

OPIUM sales have increased in Georgia as a consequence of prohibition.

DR. WM. BENJAMIN CARPENTER, the English physiologist, died in London last month.

THE cuca plant from which the new local anæsthetic is obtained, is reported as being planted on a large scale in Ceylon.

OUR friend, Frank Sage, edits the dental department of the *Cincinnati Medical and Dental Monthly*. Frank ought to make it a "go."

---

"PROF. KOEBERLE, of Strasbourg, has received a fee of $100,-000 from a Spanish prince for professional attention." That prince ought to have Dr. Atkinson fill a tooth for him.

---

BORN.—To Mrs. Wallace Searles, November 14th, 1885, a daughter. Mrs. Searles is daughter to Dr. A. O. Rawls, of Lexington, Ky.; grandpa has the congratulations of many friends.

---

A BOTTLE of bromine with the stopper out, left in a closed room over night, will destroy infection as well as insect life. It is thought to be more effectual than the fumes of burning sulphur.

---

DR. JOHN M. RIGGS, of Hartford, Conn., famous for his treatment of what was formerly known as "Rigg's Disease," died November 11th; his will directed his remains to be cremated.

---

THE January number of the REGISTER will contain the papers and discussions of the meeting of the State Society, at Chillicothe, the present number having the business of organization, Constitution, By-Laws, etc.

---

COCAINE is already being adulterated. Professor Kastenbine, reports the *Medical Herald*, analyzed a preparation sold by a Detroit chemist, and found it to contain two-thirds of boric acid. —*New York Medical Record.*

---

A PHILADELPHIA lady says that if you will trim your finger-nails every Friday you will never have the toothache. She has practiced it for over twenty years, and it has never failed.

N. B.—She wears store teeth.—*Boston Post.*

---

BUSCHONG, the famous catcher for the St. Louis Browns, Base Ball Club, is a dentist by profession and is the means of doing much good among the ball fraternity by attending to their teeth. Dr. Carver, the rifle shot, is also a dentist; thus do we become known!

---

NITRO GLYCERINE appears to be becoming a favorite remedy for many ills. It may not be long before dentists will learn to use it with effect in obstinate cases of many kinds, and then folks will learn that it is not always safe to "blow up" the dentist when a filling drops out.

---

J. C. MACKENZIE, M. D., of Cincinnati, says that the odor of Iodoform may be disguised by adding five grains of Coumarin to one ounce of Iodoform and mixing thoroughly. Coumarin is the active principle of the Tonka bean, and has the effect of changing the odor of the Iodoform to that of the bitter almond or Hydro Cyanic acid, which is not at all disagreeable. An ethereal solution of the mixture may also be made if desirable.

---

WE recommend to our readers, and especially to students, some remarks by Professor O. A. Wall, of St. Louis College of Pharmacy, on "Photography and Lantern Slides." They appear in the *Pharmaceutical Record* for November 1st.

Illustrations for lectures add so much to the effectiveness of teaching that no means should be neglected to increase the resources of our colleges in this respect.

While some of the colleges and the professors have gathered a great many slides, there are some subjects for which it is difficult to obtain illustrations.

It would be desirable that such as take an interest in the welfare of their alma mater, should occasionally photograph what would be of interest to all students.

If the student or graduate did this through enthusiasm and love for his alma mater, he would have the consciousness of having helped it materially in its task of reaching future generations.

ECHOES OF THE MEETING AT CHILLICOTHE.

"All right" is a good hotel manager.

Dr. A. Berry's paper was not read for want of time, it will be published.

The Council Chamber at Chillicothe is one of the best halls the State Society has ever met in.

Dr. Jennings, of Cleveland, was the first one elected after the organization was completed.

There were a few "postage stamps" licked one night, not many though; through a glass darkly as it were.

It was recommended that hereafter all warm fomentations shall not be applied in colore l cloths, white muslin is the proper caper.

It is a subject of congratulation for the members to know that hereafter but little time will be required for the transaction of routine business.

Kentucky sent three visitors to the meeting, Drs. Rawls, Van Antwerp and Justus, all of whom were elected to honorary membership. Come again, gentlemen.

Dr. Rehwinkel made everything so pleasant and agreeable for the members that they almost concluded to hold the next meeting at Chillicothe; Toledo, however, took the prize.

The Society will publish no transactions of the past meeting, they will be found in the Register, so that all wishing to complete their files would do well to procure extra numbers.

The report of the discussion on Pyorrhœa Alveolaris to be printed in the January number, will be very interesting, as it was participated in by Drs. Rehwinkel, Rawls and Harlan, all famous on Pyorrhœa.

In introducing Dr. Callahan, re-elected Secretary, Dr. Butler said the efficiency of the presiding officer largely depended upon the character of the Secretary who "backed him up," and that the Ohio Society has in Dr. Callahan one of the best Secretaries in the country. Correct.

WHEN it was announced that it was time to sign the Articles of the Constitution, Dr. Rehminkel stepped out of the room and returned in a few minutes with the janitor, bearing a seedy looking old table. Several of the members looked at it with surprise and suspicion, wondering that such an old trap should be brought into a Chamber so nicely fitted up. Surprise and distrust vanished from every face when Dr. Rehwinkel said the old table was a relic of early days, being the identical table upon which the first Constitution of the State of Ohio was signed by the hardy backwoodsmen in 1802, Ohio having been up to that time a portion of the North-West Territory. The top of the table is oval, made of several very thick black walnut boards dovetailed upon a frame of pine, the legs let into the top. The edges are hacked and hewed, deep notches appearing here and there, all polished by constant use and the lapse of time. The Doctor thought it was proper that the Constitution of the re-organized and incorporated State Dental Society should be signed upon it for the sake of the sentiment attached; his speech was received with a cheer and a round of applause, each one feeling that his visit to Chillicothe had not been in vain.

